# Prognostic value of normal and non-obstructive coronary artery disease based on CT angiography findings. A 12 month follow up study

**DOI:** 10.15171/jcvtr.2019.52

**Published:** 2019-10-24

**Authors:** Amirreza Sajjadieh Khajouei, Atoosa Adibi, Zahra Maghsodi, Majid Nejati, Mohaddeseh Behjati

**Affiliations:** ^1^Al-Zahra hospital, Isfahan University of Medical Sciences, Isfahan, Iran; ^2^Anatomical Sciences Research Center, Kashan University of Medical Sciences, Kashan, Iran; ^3^Rajaie Cardiovascular Medical and Research Center, Iran University of Medical Sciences, Tehran, Iran

**Keywords:** Computed Tomography, Angiography, Coronary Disease, Prognostic Value

## Abstract

***Introduction:*** The advent of multi-slice computed tomography (CT) technology has provided a new promising tool for non-invasive assessment of the coronary arteries. However, as the prognostic outcome of patients with normal or non-significant finding on computed tomography coronary angiography (CTCA) is not well-known, this study was aimed to determine the prognostic value of CTCA in patients with either normal or non-significant CTCA findings.

***Methods:*** This retrospective cohort study was performed on patients who were referred for CTCA to the hospital. 527 patients with known or suspected coronary artery disease (CAD), who had undergone CTCA within one year were enrolled. Among them, data of 465 patients who had normal (no stenosis, n=362) or non-significant CTCA findings (stenosis <50% of luminal narrowing, n=103) were analyzed and prevalence of cardiac risk factors and major adverse cardiac events (MACE) were compared between these groups. In addition, a correlation between these factors and the number of involved coronary arteries was also determined.

***Results:*** After a mean follow-up duration of 13.11±4.63 months, all cases were alive except for three patients who died by non-cardiac events. Prevalence of MACE was 0% and 3% in normal CTCA group and non-significant groups, respectively. There was no correlation found between the number of involved coronary arteries and the prevalence of MACE (*P* = 0.57).

*** Conclusion:*** A normal CTCA could be associated with extremely low risk of MACE over the first year after the initial imaging, whereas non-significant obstruction in coronary arteries may be associated with a slightly higher risk of MACE.

## Introduction

Coronary artery disease (CAD) is the leading cause of death in the industrialized world.^1,2^ Although catheter coronary angiography has been considered as the gold standard for the diagnosis of CAD, it is an expensive and invasive diagnostic method with a small (0.1-0.2%) risk of major complications such as death, myocardial infarction and stroke.^3^ Thus, it is highly desirable to find a primary non-invasive technique for the diagnosis of CAD.^4^ The advent of multi-slice computed tomography (CT) technology has provided a new promising tool for non-invasive assessment of the coronary arteries.^5^ Several studies have been performed to evaluate the diagnostic performance of CT coronary angiography (CTCA) for the detection of significant CAD.^6,7^

Some investigations have reported excellent accuracy for multi-slice CTCA compared with conventional coronary angiography,^8,9^ suggesting a potential role for this technique as an alternative first-line imaging modality appropriate for the diagnosis of patients suspected of having CAD.^10,11^ In addition to non-invasiveness, CTCA has another major advantage of depicting not only the lumen but also the wall of the coronary arteries. Therefore, it can determine whether the plaque is calcified or non-calcified and whether there is any associated positive or negative vascular remodeling.^12^ It is important for both patients and clinicians to know how accurate CTCA detects or rules out CAD. Although CTCA seems to be a useful modality, limited data are available to support the prognostic value of this diagnostic technique.^13^

Some CTCA studies showed that patients with severe CAD such as significant stenosis or multi-vessel disease had a higher risk of a worse outcome. However, the prognostic outcome of patients with normal findings or non-significant atherosclerotic plaques on CTCA is not well-known.^14^ Therefore, this study was designed to determine the prognostic value of CTCA in patients with either normal or non-significant CTCA findings.

## Materials and Methods

This retrospective cohort study was performed on patients who were referred for CTCA to the multi-detector CT scan unit of Al-Zahra hospital, Isfahan, Iran, between March 2010 and March 2011. All CTCA studies were performed with a multi-detector 64-slice CT scanner (General Electric). About 527 patients with known or suspected CAD, who had undergone CTCA within last year were enrolled. Among them, data of 465 patients who had normal (no stenosis, n = 362) or non-significant CTCA findings (stenosis <50% of luminal narrowing, n = 103) were analyzed and prevalence of cardiac risk factors and major adverse cardiac events (MACE) were compared between these groups.^15^ According to the number of involved coronary arteries, patients with non-significant coronary obstruction were subdivided to a single coronary vessel, two-coronary-vessel, and three-coronary-vessel involvement. Those who were not available for follow-up were excluded. In addition to the demographic information, data about patients’ symptoms and risk factors at the time of admission were extracted from their files.

### Follow-up

A telephone interview was arranged to collect information about the patients’ condition after CTCA. A standardized questionnaire was used to ask patients regarding any MACE including unstable angina, myocardial infarction, revascularization and death after the CTCA. If there was any history of the aforementioned events, a direct interview was arranged, and patients were asked to bring all associated medical records to be reviewed by the investigator. Three patients died at the time of follow-up due to non-cardiac causes (bladder cancer, aorta aneurysm, and car accident). Their cause of death was verified by an official death certificate.Finally, the analysis was performed on the remaining 465 subjects.

### Statistical analysis

Data were analyzed by SPSS 20 using Spearman correlation, ANCOVA, Mann-Whitney, Fisher’s exact test, and chi-square. *P* values less than 0.05 were considered statistically significant.

## Results

### Baseline characteristics

There was no significant difference between groups in terms of the prevalence of hyperlipidemia, hypertension, family history of CAD and Smoking but those with non-significant CAD were older and more male than female and diabetes mellitus was more prevalent than normal group (29% vs. 15%). Demographic data are presented in Table 1. From all 465 participants, 387 patients had a stress exercise test performed. There was no significant difference between groups regarding the results of the exercise test (Table 2).

**Table 1:** Baseline characteristics of participants

	**Non-significant (n=103)**	**Normal (n=362)**	**Total (n=465)**	**OR**	**CI**	***P*** **-value**
Age (year)	64.28±9.92	53.45±11.16	55.83±11.78			<0.0001
Sex (M/F)	52/51	121/241	173/292	1.88	(1.21, 2.93)	0.004
DM	30 (29%)	54 (15%)	84 (18%)	2.39	(1.43, 4.01)	0.001
HTN	53 (51%)	162 (45%)	215 (46%)	1.35	(0.86, 2.10)	0.11
HLP	53 (51%)	157 (43%)	210 (45%)	1.42	(0.91, 2.22)	0.07
FH	55 (53%)	182 (50%)	237 (51%)	1.16	(0.75, 1.82)	0.28
Smoking	14 (13%)	28 (8%)	42 (9%)	1.89	(0.95, 3.75)	0.053

Data are presented as mean ± SD, number or number (%). DM: diabetes mellitus, HTN: hypertension, HLP: hyperlipidemia, FH: family history of CAD, OR: odds ratio, CI: confidence interval

**Table 2:** Distribution of different stress exercise test results between groups

**Result**	**Non-significant** **(n=76)**	**Normal** **(n=311)**	**Total** **(n=387)**	***P*** **value**
Negative	40 (53%)	128 (41%)	168 (43%)	0.09
Positive	32 (42%)	145 (46%)	177 (45%)	
Suspicious	4 (5%)	38 (12%)	42 (11%)	

### The number of involved coronary vessels and risk factors

There was a meaningful difference between men and women regarding the number of involved coronary arteries with significantly more men with three-vessel involvement (Table 3). There was a strong positive correlation between age and number of involved coronary vessels (r: 0.39, *P* <0.001).

**Table 3:** Sex distribution of number of involved coronary arteries (ICA)

**Number of ICA**	**Male**	**Female**	**Total**	***P*** **value**
0	121 (33%)	241 (67%)	362 (100%)	<0.001
1	26 (47%)	29 (53%)	55 (100%)	
2	9 (45%)	11 (55%)	20 (100%)	
3	17 (60%)	11 (40%)	28 (100%)	
Total	173 (37%)	292 (63%)	465 (100%)	

After controlling for age as a covariate, there was no significant correlation between the number of involved coronary vessels and diabetes (*P* = 0.45), HTN (*P* = 0.86) and HLP (*P* = 0.40). Positive FH was significantly more prevalent in patients with non-significant involvement of two-coronary arteries (*P* = 0.03), and smoking and positive stress exercise test were significantly more prevalent in patients with non-significant involvement of three-coronary arteries (*P* = 0.01 and 0.03 respectively).

### Follow-up data

The mean follow-up duration was 13.11±4.63 months, 13.05±4.87 months in the non-significant group vs. 13.18±4.50 months in the normal group, (*P* = 0.80). Except for 3 patients who died due to non-cardiac events, all of the participants were alive after one year of follow-up. No MACE was found in participants with normal CTCA findings. Three cases (3%) in non-significant CAD group had MACE. Considering the overlaps of cardiac events, one patient (1%) had an acute myocardial infarction, two patients (2%) had experienced unstable angina, and three patients (3%) had undergone revascularization over the follow-up period (*P* = 0.22, 0.04 and 0.01respectively). However, after controlling for age, there was no correlation between the number of involved coronary vessels and MACE (*P* = 0.57) including myocardial infarction, unstable angina, and revascularization (*P* = 0.44, 0.50 and 0.67, respectively).

## Discussion

CTCA is a rapidly emerging noninvasive diagnostic tool for the detection of CAD. It has been demonstrated that this diagnostic tool has good characteristics for diagnosis of CAD, and maybe reduced the need for invasive coronary angiography.^16^ Risk stratification plays a significant role in the diagnosis and management of patients who present with chest pain with possible ischemic origin.^17^ Based on CTCA records, physicians could reassure the low-risk patients and refer the high-risk patients for further evaluation. Unfortunately, most of these patients are at medium risk with poor prognosis and subsequent treatment.

The ability to further differentiate these patients from low-risk patients after conventional risk classification is one of the most important goals of noninvasive imaging techniques. Patients at moderate risk require additional testing with one or more noninvasive procedures, such as exercise ECG, stress echocardiography, or stress myocardial perfusion imaging. Limited data are available regarding the prognostic value of CTCA, especially when the results are normal or non-diagnostic. This lack of robust outcome from CTCA data limits its role in the clinical guidelines.^11^

We found that none of the patients with a normal or non-significant CTCA died during follow-up for cardiac reasons. Furthermore, when the CTCA was normal, none of the participants developed MACE for at least one year after CT scanning. In contrast, patients with non-significant CTCA findings showed a relatively small risk for development of MACE (unstable angina, myocardial infarction, and revascularization). The prevalence of MACE in the non-significant group was higher than the normal group. There was no significant correlation between the number of involved coronary arteries and the prevalence of these events. These key findings imply that having a normal CTCA could be suggestive of an uneventful 1-years outcome. On the contrary, having some plaques on CTCA, regardless of the number of involved coronary arteries, could be associated with slightly higher rates of major cardiac problems over the first year after the imaging. Previous studies mostly compared normal CTCAs with patients who had coronary stenosis on CTCA, and hence, there are only a few data regarding the prognostic value of non-significant CAD on CTCA. According to Russo et al, the presence of CAD was associated with increased risk for hard cardiac events, which was higher in patients with significant coronary stenosis and negligible in subjects with non-obstructive CAD disease, comparable to our results.^18^

Min et al discussed that presence of plaque would successfully identify patients at risk for MACE, and negative CTCA would portend an extremely low risk for cardiac problems.^19^ Schlett et al reported a 2-year MACE free warranty period in the absence of CAD findings by CTCA.^14^ Reimann et al found that although significant coronary stenosis (>50%) was associated with increased risk of MACE within 12–24 months, non-significant CAD on CTCA was not associated with a significant increase in risk for MACE, which was in accordance with our data.^20^ In a meta-analysis by Abdulla and colleagues, the presence of non-significant CAD in CTCA was associated with modestly increased risk of MACE, which confirms our data.^15^

The limitations of this study are the sample size of 527 cases and the short time period of follow up (13±4 months). A longer follows up and higher sample size may detect more events.

## Conclusion

We concluded that a normal CTCA could be associated with an extremely low risk of MACE over the first year after the initial imaging. But finding non-significant obstruction in even one coronary artery may be associated with a non-significantly higher risk of MACE but does not necessitate the need for strict follow-up and management.

## Competing interests

None.

## Ethical approval

All procedure of this study was approved by the Isfahan University of Medical Sciences ethics committee with code: ir.mui.rce. 1391.2.106.
